# Homocysteine and Hypertension in Diabetes: Does PPAR*γ* Have a Regulatory Role?

**DOI:** 10.1155/2010/806538

**Published:** 2010-06-29

**Authors:** Utpal Sen, Suresh C. Tyagi

**Affiliations:** Department of Physiology and Biophysics, University of Louisville School of Medicine, 500 South Preston Street, Louisville, KY 40202, USA

## Abstract

Dysfunction of macro- and microvessels is a major cause of morbidity and mortality in patients with cardio-renovascular diseases such as atherosclerosis, hypertension, and diabetes. Renal failure and impairment of renal function due to vasoconstriction of the glomerular arteriole in diabetic nephropathy leads to renal volume retention and increase in plasma homocysteine level. Homocysteine, which is a nonprotein amino acid, at elevated levels is an independent cardio-renovascular risk factor. Homocysteine induces oxidative injury of vascular endothelial cells, involved in matrix remodeling through modulation of the matrix metalloproteinase (MMP)/tissue inhibitor of metalloproteinase (TIMP) axis, and increased formation and accumulation of extracellular matrix protein, such as collagen. In heart this leads to increased endothelial-myocyte uncoupling resulting in diastolic dysfunction and hypertension. In the kidney, increased matrix accumulation in the glomerulus causes glomerulosclerosis resulting in hypofiltration, increased renal volume retention, and hypertension. PPAR*γ* agonist reduces tissue homocysteine levels and is reported to ameliorate homocysteine-induced deleterious vascular effects in diabetes. This review, in light of current information, focuses on the beneficial effects of PPAR*γ* agonist in homocysteine-associated hypertension and vascular remodeling in diabetes.

## 1. Introduction

The peroxisome proliferator-activated receptors (PPAR) are members of the nuclear receptor family of ligand-activated transcription factors that regulate gene expression [1, 2]. PPAR heterodimerizes with retinoid X receptor (RXR) and the ligand-activated PPAR binds to a specific DNA binding site, termed the PPAR response element (PPRE) [3, 4] to become transcriptionally active. There are three PPAR subtypes—PPAR*α*, PPAR*δ* (also known as PPAR*β*), and PPAR*γ*, which regulate gene expression in a variety of process, including lipid and glucose metabolism, atherosclerotic plaque formation, cellular differentiation, angiogenesis, inflammation, hypertension, and heart failure [5–7]. Although three subtypes of PPAR share many aspects of biology, each of the isoforms has specific tissue distribution, ligand selectivity, and unique biological effects [8]. PPAR*α* is highly expressed in the liver, and mainly regulates lipid uptake and fatty acid catabolism. The vascular endothelial cells play a major role in regulating vascular tone, and although endothelial cells expresses PPAR*α* [9], the role of PPAR*α* and its agonist on blood pressure is still uncertain and controversial [7]. PPAR*β*/*δ* is the most widely expressed isoform that is expressed at low levels in almost all tissues. Studies in animal models have shown that although PPAR*δ* does not have role in changing blood pressure, it does have antiatherogenic effect [10]. PPAR*γ* is expressed at the highest levels in adipose tissue, where it regulates numerous genes and improves insulin sensitivity, increases fatty acid uptake, and decreases lipolysis. It was first described as an anti-inflammatory agent, however, the expression of PPAR*γ* in vascular endothelial cells and vascular smooth muscle cells raises the possibility of its involvement in the regulation of vascular tone and blood pressure [11].

Glitazones are a class of drugs primarily used to treat type 2 diabetes and related diseases. Glitazones bind to PPAR, specifically PPAR*γ*, and activate the receptor, which in turn increases the insulin sensitivity and are clinically used to control hyperglycemia in type 2 diabetes. It is known that 65% of diabetic patients also suffer from hypertension and treatment with glitazone was also noted to lower blood pressure. Diabetic subjects also often experience renal volume retention. This is one of the mechanisms by which diabetic subjects accumulate homocysteine in the body. Interestingly, clinical research suggests that at elevated levels, homocysteine is an independent risk factor for greater mortality in type 2 diabetic patients as compared to nondiabetic subjects [12]. In animal models of type 2 diabetes, glitazone (pioglitazone) is reported to reduce tissue (but not plasma) homocysteine level resulting in decreased cardiac remodeling, contractile dysfunction, and hypertension [13]. In this review, we discuss the beneficial effects of PPAR*γ* activation on vasculature through homocysteine clearance, which leads to improvement of endothelial-dependent vascular relaxation, in addition to its known hypoglycemic activity, resulting in restoration of blood pressure in diabetic nephropathy.

## 2. Renal Mechanism of Hypertension in Diabetes

In diabetes, progressive renal failure leads to end-stage renal disease [14]. Increased urinary albumin excretion, decline glomerular filtration rate (GFR) and high blood pressure are the hallmarks of diabetic nephropathy [15]. These renal functional changes during diabetes develop as a consequence of structural abnormalities and changes in podocytes. Impaired autoregulation of glomerular filtration rate (GFR) in diabetic kidney raises the blood pressure in the glomerular microcirculation [16]. Structural abnormalities including glomerular basement membrane thickening, mesangial expansion, extracellular matrix accumulation leads to glomerulosclerosis and interstitial fibrosis [17]. This raises blood pressure in the renal microcirculation and over time, uncontrolled high blood pressure can even further damage the blood vessels and nephrons causing renal volume retention and sodium accumulation in diabetes. These extra fluids and sodium linger in the bloodstream, putting extra pressure on the walls of the blood vessels, and raises the blood pressure.

## 3. Hypertension-Associated Renal Complications in Diabetes

Sustained elevation of blood pressure amplifies diabetic complications within the glomerulus by inducing impairment of autoregulation of the microcirculation, resulting in an increase in intraglomerular capillary pressure [17]. The changes of capillary pressure are paralleled by changes in overall glomerular volume [18, 19] and cyclic changes in glomerular volume lead to recurrent episodes of stretch and relaxation of all the glomerular component, including mesangial cells [19] and podocytes [20]. In vitro experimental evidences suggest that cyclic stretch/relaxation episodes in mesangial cells lead to production of extracellular components such as collagen [21], increases expression of profibrotic transforming growth factor-*β*1 (TGF-*β*1) [22], enhances the expression of the cytokine monocyte chemoattractant protein-1 (MCP-1) [23] and the cell adhesion molecule intercellular cell adhesion molecule-1 (ICAM-1) [24]. These molecules mediate and/or amplify renal damage [17]. In addition, accumulation of plasma homocysteine in diabetic nephropathy further contributes to renal damage and hypertension-associated renal complications [25, 26].

## 4. Renal Insufficiency, Homocysteine Accumulation, and Hypertension

**Figure 1:**
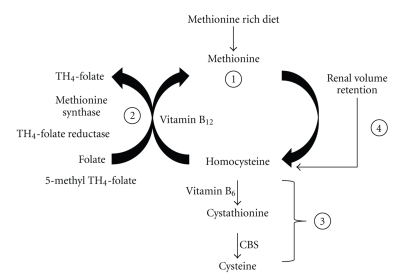
Schematic of homocysteine accumulation in the body.

Homocysteine is a nonprotein amino acid and metabolite of methionine. Homocysteine can be recycled into methionine; however, dysregulated methionine metabolism leads to accumulation of plasma homocysteine levels termed as hyperhomocysteinemia (HHcy). HHcy is an independent vascular risk factor and plasma homocysteine increases during renal insufficiency [27, 28]. There are four ways by which homocysteine can accumulate in the plasma. These are (1) a methionine-rich diet, such as meat, (2) deficiency of vitamin B_12_/folate, (3) deficiency of CBS activity (heterozygous or homozygous, CBS+/− or CBS−/−) and vitamin B_6_, and (4) renal insufficiency causing volume retention (Figure 1). Herein, we discuss how renal insufficiency and impaired glomerular filtration can cause accumulation of plasma homocysteine, which may contribute to hypertension. 

Elevated level of plasma homocysteine has always been associated with patients exhibiting chronic kidney diseases, especially end-stage renal disease (ESRD) and the prevalence of HHcy is strongly associated with decreased glomerular filtration rate (GFR) [29]. Although the precise mechanism by which GFR is related to plasma homocysteine concentration is not well established, the association of plasma homocysteine and GFR has been shown to be linear [30, 31], with increase in plasma homocysteine level corresponding to a greater decline of GFR [32]. Thus, the association between hyperhomocysteinemia and renal failure may be causal where renal dysfunction increases plasma homocysteine level. There are two different hypotheses proposed for homocysteine accumulation during renal dysfunction [29]. These are (1) homocysteine clearance is disturbed in the failing kidney; (2) extrarenal homocysteine metabolism is impaired during renal failure. These are discussed below. 

### 4.1. Homocysteine Metabolism and the Failing Kidney

The kidney is capable of filtering homocysteine, as it does for other amino acids. However, the amount of filtered homocysteine found in urine is minimal (6 *μ*mol/day, which is 1%), suggesting that most of the (99%) filtered homocysteine is reabsorbed by the kidney. The location of this uptake is reported to be on the basolateral tubule cell surface [33]. The kidney contains both transulfuration (cystathionine *β*-synthase and cystathionase) and remethylation (methionine synthase) enzymes in human [29, 33] and rats [34], which indicate that theoretically both enzymatic pathways can be used. The in vitro and in vivo studies in rat however suggest that homocysteine is primarily metabolized by transulfuration pathway (Figure 1) to form cystathionine, which is further split into cysteine and *α*-ketobutyrate [35, 36]. It is hypothesized that the kidney compensates the changes in GFR by up- or downregulating the biochemical pathways of homocysteine metabolism, thereby keeping the constant amount of homocysteine in the urine of normal healthy subjects [30]. As renal function declines during ESRD, plasma homocysteine level increases and the vast majority of dialysis patients experience mild-to-moderate hyperhomocysteinemia [37]. Studies have demonstrated inverse relationship between homocysteine and renal function [30, 33], and powerful indirect evidence suggests that elevated plasma homocysteine levels in renal disease are intimately associated with kidney function [33].

### 4.2. Renal Failure and Extrarenal Homocysteine Metabolism

Studies using a stable isotope method of whole body sulphur amino acid metabolism in ESRD patients and healthy subjects conducted by the research group led by van Guldener et al. [38–40], report that total remethylation and transmethylation flux were decreased in ESRD patients without any change in transulfuration rate as compared to control subjects. Based on their findings, they suggested two possible mechanisms that could explain elevated plasma homocysteine level in ESRD. These are (1) a defect in the sulfur amino acid metabolism that would lead to accumulation of homocysteine, and/or (2) a defect in homocysteine remethylation, which eventually increases the level of homocysteine. In any or both the cases homocysteine will be accumulated in the body due to impaired metabolism.

## 5. Homocysteine and Hypertension

The concerns are “is hyperhomocysteinemia associated with hypertension; if so, is this relationship causal; and if that is the case, does PPAR*γ* activation prevent this change?” At present, it does not appear that there is sufficient affirmative literature on these topics. However, the hypothesis that homocysteine may play a role in the pathogenesis of essential hypertension is based on the fact that homocysteine induces arteriolar constriction, renal dysfunction and increased sodium reabsorption, and increases arterial stiffness [41, 42]. Also, elevated homocysteine is known to increase oxidative stress that causes oxidative injury to the vascular endothelium, diminishes vasodilation by nitric oxide, stimulates the proliferation of vascular smooth muscle cells, and alters the elastic properties of the vascular wall [43]. All these are associated with the rise in hypertension. Thus, homocysteine may contribute to blood pressure elevation.

## 6. Diabetic Nephropathy and Homocysteine Clearance: The Role of PPAR*γ*

**Figure 2:**
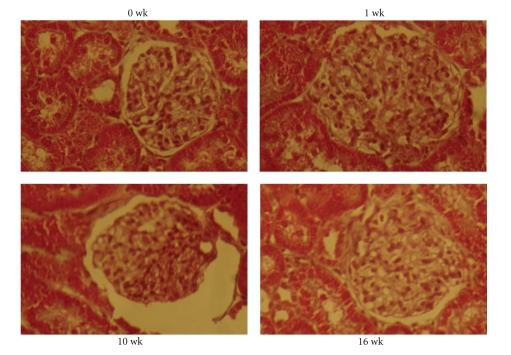
*Glomerular hypertrophy and collapse in diabetes were ameliorated by ciglitazone.* Histological kidney section were stained with Masson-Trichrome stain and visualized under dissecting microscope. Note that glomerular hypertrophy was observed at one week of alloxan (a single dose of 65 mg/kg body wt intraperitoneally) treatment. At 10 weeks glomerulus was collapsed. Ciglitazone treatment after 10 weeks of alloxan treatment reversed glomerular deformation towards normal (magnification, x200).

**Figure 3:**
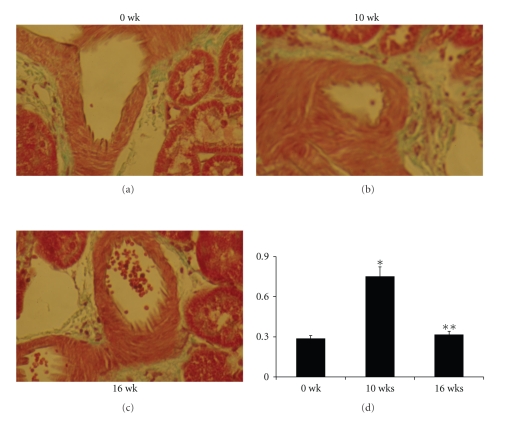
*Increased media-lumen ratio of preglomerular arteriole and tubule of diabetic mice were normalized with ciglitazone treatment.* Kidney sections of 0 wk (a), 10 wk of alloxan treatment (b), and 10 wk of alloxan treatment followed by another 6 wk of CZ treatment (c) were stained with Masson-Trichrome. (d) Preglomerular arterioles of these stained sections were identified under a microscope, and medial/lumen ratio was calculated by a digital micrometer and plotted (data  presented ± SE, *n* = 6 animals/group; *P < .01 compared with 0 wk; **P < .05 compared with 10 wk). The results indicated that medial/lumen ratio was increased dramatically due to thickening of the media and narrowing of the lumen after 10 wk of alloxan treatment. Interestingly, ciglitazone treatment almost normalized the media/lumen ratio indicating the involvement of PPAR*γ* in this process.

Diabetes mellitus, a chronic metabolic disorder, is associated with increased risk of cardio-renovascular diseases such as arterial disease, stroke, and nephropathy [44, 45]. Diabetic nephropathy (DN) is a leading cause of morbidity and mortality in hyperglycemic patients and the most common single condition found in end-stage renal disease (ESRD) [46]. The majority of diabetic patients with renal failure suffer from glomerulopathy which is characterized by glomerulosclerosis, increased thickness of the glomerular basement membrane, glomerular hypertrophy, mesangial cell expansion, podocytic loss, and tubulointerstitial fibrosis leading to progressive reduction of glomerular filtration rate (GFR) [46, 47]. Chronic diabetes reduces PPAR*γ* mRNA level in the glomeruli [48] and in the pathogenesis of DN downregulated PPAR expression is associated with matrix accumulation, such as collagen IV and glomerulonephritis [49–52]. Activation of PPAR*γ* regulates gene expressions that promote insulin sensitization and glucose metabolism [53]. In addition, several studies have demonstrated the efficacy of PPAR agonists to inhibit the progression of glomerulosclerosis [54] and have suggested that PPAR ligands have a direct beneficial renal effect. For example, in experiments on diabetic rats with nephropathy, treatment with PPAR*γ* agonist reduced the occurrence of albuminuria and prevented the development of glomerulosclerosis and glomerular hypertrophy (Figure 2) by suppressing TGF-*β*, VEGF, PAI-1, collagen IV, and ICAM-1 [55, 56]. We have reported that PPAR*γ* agonist ciglitazone improved GFR and glomerular architecture in diabetic nephropathy, in part, by normalizing tissue levels of homocysteine in the glomerulus [25]. Impairment of renal function, as evidenced by reduced GFR was noticed due to vasoconstriction of glomerular arteriole (Figure 3), which resulted renal volume retention and increased plasma homocysteine levels [57]. Elevated plasma homocysteine, in turn, caused chronic and impaired renal filtration and was also reported as a risk factor for diabetic nephropathy [58, 59]. Activation of PPAR induced insulin sensitivity in type 2 diabetes and promoted tissue uptake of homocysteine; these resulted in lowering of plasma homocysteine levels [57, 60]. Contrary to this mechanism in type 1 diabetes the plasma homocysteine level did not change, although increased glomerular tissue level of homocysteine became normal with CZ treatment [25]. We suggested that this change of tissue homocysteine level was probably because of improvement of diabetic nephropathy that normalized renal volume retention and accelerated the clearance of glomerular tissue homocysteine. This finding was in accordance with the clinical trials where PPAR agonists ameliorated endothelial dysfunction in hyperhomocysteinemia (HHcy) with no effect on plasma homocysteine level [61].

## 7. Homocysteine, Matrix Remodeling, and Hypertension: The Role of PPAR*γ*

**Figure 4:**
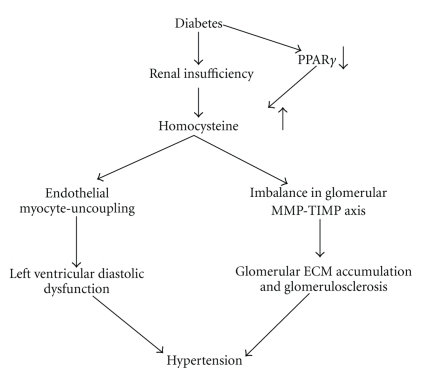
*Proposed mechanism of homocysteine associated hypertension in diabetes.* Diabetes causes renal microvascular constriction and deposition of extracellular matrix in the glomerular basement membrane. This causes glomerulosclerosis and impaired glomerular function (GFR). Renal hypofiltration increases plasma homocysteine level, which further cause oxidative stress and amplifies glomerular injury. Increased matrix accumulation in the myocardium leads to deposition of extracellular matrix between endothelium and myocyte causing endothelium myocyte uncoupling. This causes prevention of NO to pass through the matrix barrier and impairs left ventricular diastolic dysfunction. Glomerulosclerosis and L-V diastolic dysfunction results in hypertension.

Extracellular matrix (ECM) plays an important role in maintenance of tissue architecture and normal physiological function. Remodeling of extracellular matrix (ECM) is a dynamic process and excessive ECM deposition is a pathophysiological phenomenon of diseased condition that could lead to hypertension [62–64]. A number of enzymes engage in the regulation of ECM turnover. Among these are MMPs and their natural inhibitor, TIMPs. MMPs are members of a family of Zn^2+^- and Ca^2+^-dependent endopeptidases, which are essential for tissue remodeling in both physiologic and pathophysiologic conditions. MMP enzymes in the normal physiologic condition reside in the latent form and are activated by various physiological threats [60]. Among MMPs, MMP-2, and MMP-9 are gelatinases that degrade collagen IV and are essential in maintaining the integrity of the glomerular basement membrane. Because the turnover of collagen is faster that gelatin, oxidatively modified collagen deposits in the tissue causing fibrosis. In diabetic nephropathy activities of MMPs and TIMPs mostly regulate ECM degradation [65]. Type IV collagenases, MMP-2 and -9, have been studied extensively in various glomerular diseases with conflicting results [57, 65–67]. We have shown previously that increases in glomerular homocysteine and activation of MMP-2 are associated with glomerulosclerosis [57]. It was, however, unclear how MMPs and TIMPs are involved in glomerulosclerosis and whether PPAR, in part, regulates these enzymes that modulate glomerular dysfunction in DN. Recently, we reported that both MMP-2 and -9 activities were increased significantly in diabetic kidney [25], and this result was in accordance with the similar findings reported by independent laboratories, including our own [57, 63, 68, 69]. We also showed that expression of TIMP-1 was upregulated in the glomeruli of diabetic mice [25], which was in agreement with the previously reported study by Eddy et al. [70] where progressive renal fibrosis was characterized by upregulation of TIMP-1 expression. At the onset of diabetes, the kidney grows larger, but it eventually shrinks with reduced GFR, proceeding to sclerosis and renal failure. We have reported that subnormal GFR was noticed at the latter stage of alloxan-induced diabetes in mice, and increase in renovalcular resistance was accompanied by collapse of preglomerular arteriole and the glomerulus [25]. This was in part due to MMP/TIMP imbalance and the accumulation of ECM matrix. PPAR agonist CZ treatment normalized these matrix proteins in diabetic kidney through activating PPAR*γ* and homocysteine clearance; thus, resulted in restoration of renal architecture, normal glomerular function, and vascular resistance of the renal arteriole [25]. A proposed mechanism of homocysteine associated matrix accumulation and hypertension has been depicted in Figure 4.

## 8. Homocysteine Handling in the Heart: The Role of PPAR*γ*

Until recently, it was our main concern to control systolic blood pressure and to keep this pressure as close as possible to normal level to minimize hypertension-associated morbidity and mortality. Recent studies however, have shifted our attention to diastolic hypertension which can be as harmful as systolic hypertension. A constant elevated diastolic pressure increases the risk of heart damage, brain damage, and kidney problems as well. One of the causes of diastolic hypertension is diastolic dysfunction, which demonstrates hypertrophy of the cardiomyocytes, increased interstitial collagen deposition and/or infiltration of the myocardium leading to endothelial-myocyte uncoupling. It is estimated that, although the majority of cardiac muscle is myocyte, sixteen percent of the myocardial mass is capillaries and the inner lining of the capillaries are made up endothelium [71]. The capillary endothelium is embedded in the cardiac muscle, and plays an important role in myocardial diastolic relaxation, in addition to those which myocytes contribute. Nitric oxide (NO) from the endocardial endothelium alters the contractile and relaxant properties of the heart [72]. A gradient of NO concentration, that is, high in endocardium and low in mid myocardium, has been documented [72], which suggests that there is more capillary endothelium in the endocardium than in epi- or mid-myocardium. Since capillary endothelial cells are embedded in the muscle, the contribution of endothelium to cardiac relaxation is the least studied. We have studied LV tissue function using a cardiac ring preparation in a tissue myobath and assessed the effects of hyperhomocysteinemia on myocardial endothelium-dependent relaxation [73]. In alloxan-induced diabetic mouse heart, our study demonstrated both plasma and myocardial tissue level of homocysteine increased. However, the tissue level of homocysteine was reduced by PPAR*γ* agonist (CZ)-treated diabetic mice without any alteration of plasma homocysteine level [73]. The decreased myocardial tissue level of homocysteine in diabetic heart treated with PPAR*γ* agonist was found to improve myocardial relaxation in vitro in both an endothelium dependent and independent way [73]. Endothelium dependent cardiac relaxation was measured by acetylcholine and bradykinin, where acetylcholine works through the endothelium-dependent NO generation, and bradykinin works on blood vessels through nitric oxide and endothelial-derived hyperpolarizing factor. Both factors have shown that endothelium dependent relaxation was impaired in diabetic cardiac rings [73]. Interestingly, endothelium independent vascular relaxation induced by sodium nitropruside also reduced cardiac relaxation in vitro in cardiac rings prepared from diabetic heart. This suggests that traveling of NO to the capillary smooth muscle cells was somehow impaired. This we referred to as endothelial-myocyte uncoupling, which did not allow nitropruside-generated NO to travel through the disrupted matrix between endothelium and myocyte. Thus, we have observed attenuated relaxation. However, treatment of diabetic mice with PPAR*γ* agonist CZ, normalized the relaxation of cardiac rings, suggesting the attributed role of CZ in endothelial-myocyte recoupling in diabetes [73]. This study demonstrated that tissue levels of homocysteine contributed endocardial endothelium function and PPAR*γ* activation promoted tissue clearance of homocysteine thereby improving endothelium dependent cardiac relaxation. On the other hand endothelium independent relaxation was improved in part by recoupling of endothelium and myocyte [73]. A possible mechanism of endothelium-myocyte uncoupling and hypertension in diabetes-associated hyperhomocysteinemic condition has been depicted in Figure 4.

## 9. Homocysteine, Protein Modification, and Hypertension: The Role of PPAR*γ*

Although the homocysteine is linked to blood pressure, a direct cause and effect relationship of hyperhomocysteinemia and hypertension has not been established. The mechanisms that could explain this relationship include homocysteine-induced arteriolar constriction, renal dysfunction, increased sodium absorption, increased arterial stiffness, and endothelial damage [74]. Other possible mechanisms that may be involved are (1) formation of homocysteine thiolactone and (2) protein homocysteinylation. At elevated levels homocysteine converts to homocysteine-thiolactone as a result of an error-editing function of some aminoacyl-tRNA synthetases, and the detailed mechanisms are described elsewhere [75–77]. Homocysteine-thiolactone is a reactive metabolite that causes protein *N*-homocysteinylation through the formation of amide bonds with protein lysine residues [77], which alters or impairs the protein's function [76]. *N*-linked protein Hcy (*N*-Hcy-protein) has been reported to be elevated in hyperhomocysteinemia [78–81], and has been documented to accumulate in atherosclerotic lesions in mice [82]. Protein homocysteinylation damages protein, manifests multimerization, and precipitates extensively modified proteins [76], which can cause cardiovascular diseases. For example, CBS-deficient patients have significantly high levels of plasma prothrombotic N-Hcy-fibrinogen [81], which leads to abnormal resistance of fibrin clots to lyses and contributes to increased risk of thrombosis. Thus, although presently the hypothesis that elevated homocysteine causes hypertension still remains unproven, the contributing role of hyperhomocysteinemia in the renovascular diseases, such as diabetic nephropathy to elevate blood pressure can not be ignored as substantial indirect evidence linked to hypertension during these disease processes. 

Genetic variations have been demonstrated to play an important role in determining plasma homocysteine levels. For example, sequence variation of methylenetetrahydrofolate reductase (MTHFR) gene has been shown to influence circulating homocysteine level [83], and sequence variation of amino acid 222 from alanine to valine (p.A222V) has been reported to elevate circulating concentrations of homocysteine [84]. The PPAR*γ* produces a number of isoforms which control a variety of pathways including lipid metabolism, insulin sensitivity, and inflammation [85]. Therefore, these transcription factors may play a significant role in controlling the enzymes critical for homocysteine production or metabolism. Interestingly, studies in animal models and patients have shown PPAR*γ* ligation to reduce circulating homocysteine concentration [86, 87]. Thus, the findings that the pharmacological PPAR*γ* ligands are able to reduce circulating homocysteine concentrations fit well with a role of PPAR*γ* in modulating homocysteine turnover [86, 87]. We have demonstrated that activation of PPAR*γ* in diabetic subjects reduced tissue homocysteine level and normalized systolic blood pressure [73]. Thus, it may be possible that PPAR*γ* activation reduces hypertension through reduction of homocysteine, at least in part. However, as direct link of hyperhomocysteinemia and hypertension is still not established, the issue of whether or not the reduction of homocysteine level through PPAR*γ* activation reduces blood pressure remains debatable and controversial. Future studies are needed to establish a direct cause and effect relationship between hyperhomocysteinemia and hypertension, if any. Nonetheless, it is time to speculate that hyperhomocysteinemia contributes to elevate blood pressure in the pathogenesis of renal disease, for example, diabetic nephropathy, and PPAR*γ* is an effective target molecule to regulate hypertension, at least in part, through the reduction of homocysteine, where renal insufficiency upregulates homocysteine.

## 10. Hydrogen Sulfide, Inflammation, and Hypertension: The Role of Homocysteine

Hydrogen sulfide (H_2_S) has been known for the decades as a noxious gaseous molecule with an intoxicating effect on the brain and central nervous system. Recent findings, however, reported that it is an effective molecule to regulate blood pressure [88, 89]. Endogenously, H_2_S is generated in the mammalian tissue from L-cysteine, and homocysteine is the precursor of L-cysteine. Physiologically, homocysteine is metabolized by three transulfuration pathway enzymes, cystathionine *β*-synthase (CBS), cystathionine *γ*-lyase (CSE) and 3-mercaptopyruvate sulfurtransferase (3MST). At elevated levels, homocysteine has been shown to reduce activity of CSE, thereby reducing the production of H_2_S [90]. Studies from independent laboratories reported that, at low levels, H_2_S defends organs from several pathophysiological conditions, such as oxidative stress, ischemia-reperfusion, and hypertension [88, 91, 92]. Interestingly, results from in vitro studies suggest that at low levels H_2_S decreases hydrogen peroxide (H_2_O_2_), peroxynitrite (ONOO^–^), and superoxide anion (O_2_^●–^) generation induced by homocysteine in a cell culture model [93]. 

**Figure 5:**
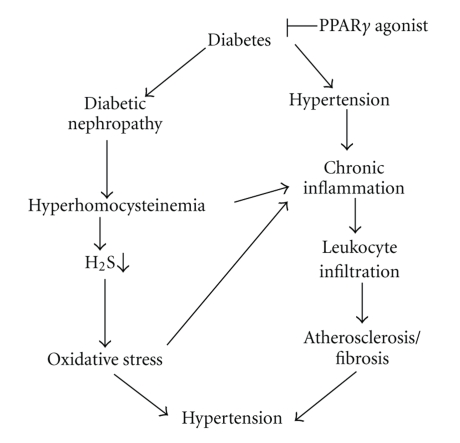
*Schematic of PPAR* *γ* *-mediated reduction in inflammatory reaction and hypertension in diabetic nephropathy.* Diabetes causes increase in homocysteine level and subsequent inhibition of hydrogen sulfide production in the body through the inhibition of cystathionine *γ*-lyase (CSE), an enzyme required for homocysteine metabolism. This leads to oxidative stress and causes hypertension. Homocysteine and diabetes induce chronic inflammation, which lead to atherosclerosis and hypertension. PPAR*γ* induction clears tissue homocysteine, in addition to regulating hyperglycemia, thereby reduces oxidative stress and hypertension.

It is known that rise in blood pressure causes chronic inflammation of the endothelium which is, in turn, responsible for further endothelial damage and worsening blood pressure. On the other hand, several metabolic disorders such as dyslipidemia, hyperhomocysteinemia, diabetes, and obesity cause inflammation followed by a subsequent rise of blood pressure. Inflammatory disease such as atherosclerosis is a major complication of hypertension [94], and plays a critical role in hypertensive renal disease, whereas treatment of renal inflammation by melatonin has been shown to ameliorate hypertension [95]. Several studies have documented that homocysteine may directly or indirectly promote synthesis of several proinflammatory cytokines in the arterial wall and in the circulating cells. In particular, the expression of MCP-1 has been shown to increase in cultured human endothelial cell [96], smooth muscle cells [97], and in monocytes treated with homocysteine [98–100]. Additionally, homocysteine-thiolactone has recently been demonstrated to be more toxic than homocysteine, and possesses stronger proinflammatory properties [101]. Furthermore, homocysteine-thiolactone impairs insulin signaling, and thereby inhibits insulin-mediated glycogen synthesis [102]. We have reported that although PPAR*γ* activation did not have any effect on plasma homocysteine level, it promoted clearance of tissue homocysteine, in addition to its known action of increasing insulin sensitivity. Thus, the activation of PPAR*γ* in diabetic nephropathy modulates inflammatory reaction, at least in three different mechanisms: (1) increases insulin sensitivity and reduces plasma glucose level, therefore reduces inflammation; (2) promotes tissue clearance of homocysteine level and thus, reduces oxidative stress and inflammation; (3) normalizes CSE enzymatic activity, thereby raises the possibility of endogenous H_2_S generation, which has been documented as an anti-inflammatory and antihypertensive gaseous molecule at physiological levels [88, 103]. The possible pathways of these mechanisms are shown in Figure 5.

## 11. Recent Clinical Trials and the Homocysteine Paradox

It is well established through decades with many large prospective studies that hyperhomocysteinemia predicts increased risk of vascular events including stroke, venous thromboembolism, and death [104, 105]. Many interventional trials paradoxically, however, failed to demonstrate any clinical benefit from homocysteine-lowering therapy [106–110]. The possible reasons are explained elsewhere [111]. Briefly, hyperhomocysteinemia is a clinically important risk factor at extremely high levels. All of the recent clinical trials of homocysteine-lowering therapy have been performed in subjects with relatively mild hyperhomocysteinemia [111]. The negative outcome of these trails may indicate that mild hyperhomocysteinemia is not a causative risk factor rather it is a marker of other vascular diseases and is associated with increased vascular risk. It is also possible that homocysteine lowering therapy may produce some adverse effect that mask the clinical benefit of lower homocysteine [108]. Also, the trials were conducted after the implementation of policies that mandate the addition of folic acid to white flour, cereals, and related products in the United States. This resulted in lower homocysteine concentration among US populations [112, 113]. Moreover, in none of the trials measurement of tissue homocysteine levels was considered. Although folic acid treatment lowered plasma homocysteine levels, it may have promoted tissue uptake of homocysteine, a similar effect where insulin reduced plasma homocysteine, but increased tissue homocysteine level [60]. This increased tissue homocysteine level mimicked the clinical benefit of homocysteine lowering effect of folic acid on cardiovascular events. Interestingly, a recent report suggests that in type 2 diabetic patients, metformin reduces both folate and vitamin B12, and increases homocysteine. Conversely, rosiglitazone decreases homocysteine level in the same time period. The clinical significance of these observations is not clear and remains to be investigated [87]. Some larger trials with longer homocysteine-lowering therapy are ongoing and we should wait until the outcomes of these trials finally settle the debate. Nevertheless, the kidney plays a major role in homocysteine metabolism and plasma homocysteine increases as renal function declines.

## 12. Concluding Remarks and Perspectives

Diabetes is the most common single factor of cardiovascular and renal damage in patients with diabetes mellitus. Diabetes causes tissue accumulation of homocysteine both in cardiac and glomerular tissue. This increased tissue content of homocysteine exacerbates cardiovasculopathy and nephropathy in diabetes, in addition to the detrimental effect of diabetes. PPAR*γ* agonists may be beneficial in preventing vasculopathies in cardiac and renal tissues associated with increased homocysteine content in diabetic subjects. Moreover, PPAR*γ* ligand seems to be promising in preventing hypertension associated with increased homocysteine level in diabetes. Although at present it is premature to conclude homocysteine causes hypertension, there is substantial indirect evidence which supports homocysteine-associated rise in blood pressure. Further studies are needed to elucidate the contributing role of homocysteine to regulate blood pressure, and precise mechanism of hypertension modulation associated with hyperhomocysteinemia by PPAR*γ* induction warrants special attention.

## Abbreviations

CBS: Cystathionine-*β*-synthase
CSE: Cystathionine-*γ*-lyase
CZ: Ciglitazone
DN: Diabetic nephropathy
ECM: Extracellular matrix
ESRD: End-stage renal disease
GFR: Glomerular filtration rate
Hcy: Homocysteine
HHcy: Hyperhomocysteinemia
H_2_S: Hydrogen sulfide
H_2_O_2_: Hydrogen peroxide
ICAM-1: Intercellular cell adhesion molecule-1
LV: Left ventricle
MIP-2: Macrophage inflammatory protein 2
MMP: Matrix metalloproteinase
NO: Nitric oxide
O_2_^●–^: Superoxide
ONOO^−^: Peroxynitrite
PAI: Plasminogen activator inhibitor
PPAR: Peroxisome proliferator-activated receptor
PPRE: PPAR response element
RXR: Retinoid X receptor
TGF-*β*: Transforming growth factor-*β*
TIMP: Tissue inhibitor of metalloproteinase
VEGF: Vascular endothelial growth factor.
